# Analysis of the Urban Land Use Efficiency in the New-Type Urbanization Process of China’s Yangtze River Economic Belt

**DOI:** 10.3390/ijerph19138183

**Published:** 2022-07-04

**Authors:** Liu Yang, Bingyang Han, Zhili Ma, Ting Wang, Yingchao Lin

**Affiliations:** 1 School of Management Science and Real Estate, Chongqing University, Chongqing 400045, China; yyangliu@cqu.edu.cn (L.Y.); hanbingyang@cqu.edu.cn (B.H.); 2 School of Public Affairs, Zhejiang University, Hangzhou 310058, China; linyc@zju.edu.cn

**Keywords:** urban land use efficiency, new-type urbanization, SE-SBM model, Sys-GMM

## Abstract

The accelerated urbanization process in China has caused a shift in the urban land use structure. The Chinese government has issued ‘the National New-type Urbanization Plan’ focusing on the rational use of resources, which is of great significance for the intensification and sustainability of land use. In promoting the construction of the new-type urbanization (N-TU), enhancing the urban land use efficiency (ULUE) is crucial to regional coordinated development. This study uses panel data from 2011 to 2020 for 11 provinces (cities) in the Yangtze River Economic Belt (YREB) and adopts the super efficiency (SE) slacks-based measure (SBM) model with undesirable outputs and the entropy weight method to calculate the ULUE and N-TU levels. The study uses the System generalized method of moment (Sys-GMM) to study the N-TU’s impact on the ULUE empirically. The results indicate: (i) the overall trend of new-type urbanization level is gradually increasing and has the characteristics of uneven spatial distribution between provinces. (ii) The ULUE shows a fluctuating upward trend during the studied period. (iii) The N-TU and its subsystems have significant positive effects on the ULUE. Overall, this study aims to explore the relationship between the N-TU and ULUE enriching the theoretical analysis and empirical research in related fields, thus helping decision makers in the assessment and design of policy recommendations.

## 1. Introduction

The unprecedented acceleration of global urbanization after the outbreak of the industrial revolution has caused a continuous restructuring of urban land use structures [1]. Relevant studies have shown that urban land is expanding more quickly than the population increase [2,3], and urban expansion will be primarily located in developing countries in the following decades [4]. China is the largest developing country globally and has made significant achievements in industrialization and urbanization [5,6,7]. Data from the China Statistical Yearbook [8] show that China’s urbanization rate has increased significantly, with rates of 36.22%, 49.95%, and 63.89% in 2000, 2010, and 2020, respectively. In addition, according to the China’s National Bureau of Statistics (various years) [9], the total land area zoned for urban construction reached 58,355.3 km^2^ in 2020, an increase of 7.7 times from 1981. The rapid development of traditional urbanization focusing on economic construction has made great contributions to promoting economic growth [10]. However, it has also caused a rough expansion of urban land, introducing issues such as an insufficient supply of land elements, unreasonable land use structure, and a serious waste of land resources, causing the low efficiency of land use, which is not conducive to urban development [11,12,13].

To make up for the shortcomings brought by the traditional urbanization strategy, the Central Committee of the Communist Party of China and the State Council released the National New-Type Urbanization Plan (2014–2020) [14] in 2014, which aims to improve the quality of urbanization development and pay more attention to the efficient use of urban land and an intensive and sustainable urban development model [10,15]. ULUE includes the output efficiency at the micro level and the structural efficiency of land allocation at the macro level [16]. The most widely studied aspect of ULUE is the input–output relationship at the micro level, which is characterized by the output efficiency per unit of land area [17]. Therefore, it is generally accepted that the ULUE is the result of the combination and interaction between land and other factors in the urban system [18,19]. To achieve the ambitious goal of having 70% of its 1.4 billion people living in cities by 2030 [20], China faces the enormous challenge of improving the ULUE.

Land is fundamental for social and economic activities but also for delivering soil ecosystem services, thus contributing to the functioning of urban and territorial systems across different scales [21,22]. The continuous advancement of urbanization increases the demand for land. Uncontrolled urbanization can cause a compromise through land take and soil sealing, thus worsening urban life quality. The Yangtze River Economic Belt (YREB) is a key national development strategy area with excellent geographical and development conditions [23]. In 2020, the YREB supported 42.9% of the country’s population and contributed 46.6% of China’s GDP with only 21.4% of the country’s land area, playing a pivotal role in China’s development process. In the past stage of rapid urbanization growth, the rough expansion of cities in the YREB led to the emergence of problems such as the waste of land resources, which is not conducive to improving the ULUE. At present, the growth of the urbanization rate in China is gradually slowing down, and the urbanization process has transformed from a phase of rapid increase to a stage of quality improvement. In this context, the purpose of this study is to understand the current situation and evolutionary trends of the N-TU and ULUE in the YREB and to clarify the relationship between them and the improvement path of the ULUE in the N-TU process. This is also information that decision makers urgently need to have a clear understanding of, which will help them to assess and design policy recommendations, and is of practical significance in promoting high-quality development in the YREB. Therefore, this study focuses on the following four questions:

- What is the evolutionary trend of the N-TU level?
- What is the evolutionary trend of the ULUE?
- What is the impact of the N-TU on ULUE?
- What paths can be taken to improve the ULUE in a new-type urbanization process?

Indicators are useful parameters that measure a single aspect of a phenomenon, and their aggregation facilitates the complex elaboration of information. By aggregating several indicators, an index can be synthesized, thus contributing to a comprehensive understanding of the phenomenon [24]. Therefore, to solve the above issues, this study constructed a comprehensive evaluation indicator system of the N-TU and ULUE. The entropy weight method and the SE-SBM model with undesirable outputs were utilized to calculate the N-TU level and the ULUE, respectively. Then, the dynamic panel regression model was established and empirically investigated the impact of the N-TU and its subsystems on the ULUE using the Sys-GMM. Finally, based on the analysis results, policy recommendations to enhance ULUE are proposed, which are expected to assist decision makers in evaluating and designing relevant policy recommendations. 

The remainder of the paper is divided into the following sections. Section 2 presents a review of the existing literature. Section 3 discusses the materials and methodology, containing the introduction to the study area, the analysis of the influence mechanisms and research hypotheses, the assessment methods of the N-TU and ULUE, the establishment of the regression models, and the introduction of the variables and data sources. The results are shown in Section 4, including the evolution trend of the N-TU level and the ULUE in YREB and the influence of N-TU on ULUE. Section 5 discusses the impact of the N-TU subsystem on the ULUE, presents the results of robustness tests, and puts forward corresponding policy recommendations. Section 6 gives the core conclusions and directions for further study.

## 2. Literature Review

At present, the study of ULUE and urbanization has achieved milestone results. Urbanization is an essential symbol of national modernization [25], and the urbanization process can promote the expansion of the consumer goods market [26,27] and contribute to the rapid growth of the tertiary sector, which in turn promotes the optimization and upgrading of industrial structures [28,29]. Although urbanization has promoted social progress, problems such as environmental pollution and disorderly land expansion have also emerged [30,31,32,33]. With the continuous progress of China’s development in all aspects, the N-TU is more in line with the basic national situation of China than traditional urbanization [34]. A single indicator has commonly been adopted in previous studies to estimate the urbanization level, such as the population urbanization rate [29,35,36]. Presently, scholars have started to use the entropy method to calculate the N-TU level as comprehensively and objectively as possible by constructing a comprehensive evaluation system [37,38]. For example, Yu [39] constructed a comprehensive system to evaluate the N-TU level in four dimensions: population, economic, social, and environmental, and used the entropy weight method to calculate the N-TU level. Deng [10] constructed the N-TU assessment indicator system from the perspectives of society, population, space, and economy and used the entropy weight method to calculate the N-TU level. Li et al. [40] used the entropy method to assess the N-TU level of the urban agglomeration in China in five dimensions: population, economy, space, society, and ecology.

China’s urbanization process has accelerated since the reform and opening up [41,42]. As a factor of production, urban construction land has been expanding, resulting in increasingly serious conflicts between land supply and demand, and a series of land-use problems have emerged [43,44,45]. The Chinese government has established a macro-control mechanism for land use by introducing land management policies and other means [44,46]. Most studies have concentrated on studying urban land use by analyzing the ULUE, and data envelopment analysis (DEA) and the stochastic frontier approach (SFA) are the prevalent approaches to evaluate the ULUE. For example, Deng and Gibson [47] used SFA to analyze the ULUE in Hebei Province, China. Ge et al. [48] used Bootstrap-DEA and Bootstrap-Malmquist methods to study the ULUE under a resource-based economic transition. Jiang et al. [49] used the DEA method to measure the ULUE. With the in-depth study of ULUE, more and more scholars have considered the impact of undesirable outputs. The slack-based measure (SBM), which can incorporate undesirable outputs, could be used in the evaluation of the ULUE. Zhu et al. [50] adopted the SE-SBM model to evaluate land-use efficiency in Chinese cities. Wu et al. [51] used the SBM-DEA model to evaluate the ULUE of the Yellow River basin. Tan et al. [52] used the SBM model to evaluate the urban land green use efficiency of cities in Yangtze River Delta, China, from 2004 to 2015.

Related studies have shown that there are many factors affecting the ULUE. Yu et al. [53] found that the degree of market openness and the economic development level positively affect the ULUE, but government intervention has a negative effect. Verburg et al. [54] explored the impact of ecological and social environment on land-use efficiency. Guastella et al. [55] investigated the effect of city size on the ULUE and found that larger cities were more efficient in land use management. Given urbanization’s great influence on ULUE, research on the relationship between ULUE and urbanization has gradually increased. Susannah et al. [56] examined the relationship between ULUE and urbanization. Macedo [57] found a mutual driving relationship between urbanization and ULUE. Masini et al. [58] analyzed the driving effect of different factors on the ULUE in 417 metropolitan cities in European countries through a stepwise multiple regression model. Deng [10] found that the N-TU positively affected urban land use based on 203 Chinese cities. Wang et al. [1] empirically investigated the spillover effect of the N-TU on green space use efficiency from the spatial viewpoint.

A review of the relevant literature revealed that academics have conducted substantial research on urbanization and ULUE, but the following shortcomings still exist. Firstly, previous studies have explored the relationship between urban land use and traditional urbanization, but there is a dearth of theoretical and empirical studies on the influence of the N-TU on ULUE. Secondly, previous studies have mainly constructed static panel models to investigate the effect of urbanization on the ULUE, ignoring that the ULUE has the characteristic of continuity.

To deal with these limitations, we study the impact of the N-TU on ULUE and discuss the paths of the N-TU affecting ULUE. This study has the following main contributions. First, the impact of the N-TU on the ULUE in the YREB was studied for the first time. As a key national strategic region, the YREB has an essential impact on the sustainable development of Chinese society in terms of its N-TU level and its ULUE. However, the relevant research in this area has not yet been conducted. The present study analyzes the evolution trend of the N-TU level and the ULUE in the YREB and helps the local government comprehensively and objectively understand the development status of the two. Second, a methodology is proposed to study the impact of the N-TU on ULUE. This study constructed a composite evaluation index system of the N-TU and ULUE based on relevant concepts, using the SE-SBM model with undesirable outputs and the entropy weight method to calculate the ULUE and N-TU levels. Then, we set up a dynamic panel regression model and empirically investigated the impact of the N-TU and its subsystems on ULUE using the Sys-GMM. Third, this study enriches the theoretical analysis and empirical investigation of the relationship between the N-TU and ULUE. It describes the mechanism of the impact of the N-TU on ULUE, proposes corresponding research hypotheses, and conducts empirical tests according to the theoretical analysis. We hope to explore the path that can improve the ULUE in the process of the N-TU and provide a theoretical and empirical basis for improving the ULUE of the YREB.

## 3. Materials and Methodology

Section 3 presents the materials and methodology. This section introduces the study area and data sources (Section 3.1), analyzes the mechanisms of the N-TU affecting ULUE, and presents the research hypotheses (Section 3.2). Finally, the variables and datasets of this study are introduced, and the econometric models and empirical methods are presented (Section 3.3).

### 3.1. Study Area and Date

#### 3.1.1. Study Area

**Figure 1:**
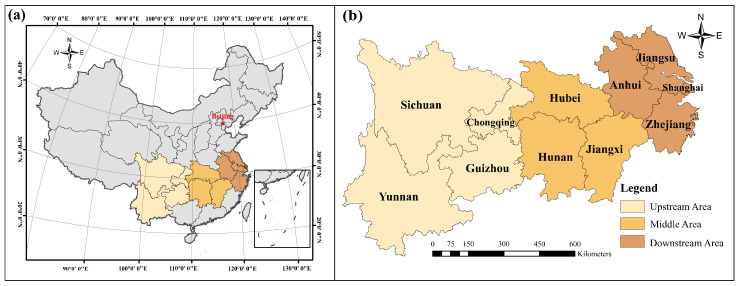
(**a**) Map of the geographical location of the YREB in China; (**b**) map of the upstream, midstream, and downstream distribution of the YREB. **Source:** Authors’ drawing using ArcGIS10.7 software.

The YREB (21°08′–35°07′ N, 97°22′–123°25′ E) is a key national strategic area with the Yangtze River as the link, spanning the three major plates of China. The YREB contains nine provinces and two municipalities directly under the Central Government, which can be divided according to upstream, midstream, and downstream of the Yangtze River. The upstream area includes Yunnan, Chongqing, Guizhou, and Sichuan provinces. The midstream area includes Hubei, Hunan, and Jiangxi provinces. Shanghai, Zhejiang, Jiangsu, and Anhui provinces are located in the downstream area. The study area of this paper is shown in Figure 1.

#### 3.1.2. Date Sources

This article investigated the impact of the N-TU on ULUE using data from 11 provinces (cities) in the YREB from 2011 to 2020. The required data were collected from the China Statistical Yearbook (2012–2021) [8], China Urban Construction Statistical Yearbook (2012–2020) [59], China Statistical Yearbook of Environmental (2012–2021) [60], and the 2011–2020 Statistical Yearbook of 11 provinces (cities) in the YREB [61]. Individual lacking data were complemented by the interpolation method. All economic indicators were transformed into comparable prices using 2011 as the base year.

### 3.2. Impact Mechanism

**Figure 2:**
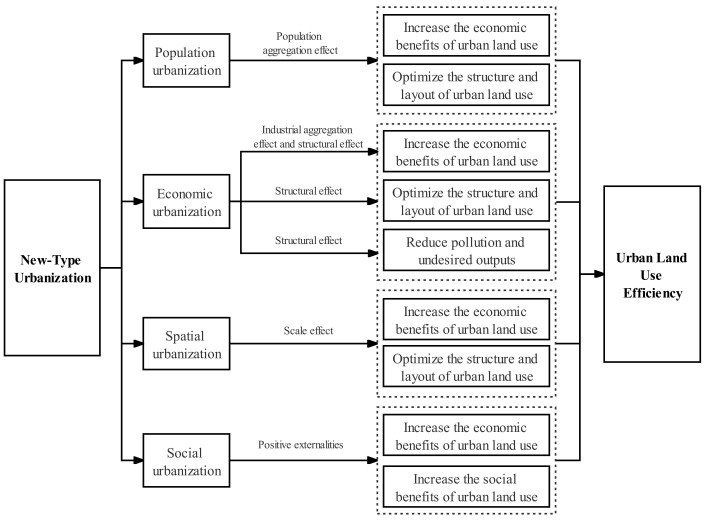
Mechanisms of the N-TU on the ULUE. **Source:** Authors’ drawing.

This study referred to the research results of Niu et al. [62], Deng et al. [10], and Han et al. [63] to analyze the intrinsic mechanism of the effect of the N-TU on the ULUE in four paths: social urbanization, economic urbanization, demographic urbanization, and spatial urbanization (Figure 2).

① In urbanization, the surplus agricultural labor force will move to non-agricultural industries after entering cities, thus optimizing factor allocation, generating economies of scale, and enhancing the economic benefits of land use. The increasing concentration of the population in cities will also put forward higher requirements for urban living, living conditions, and transportation convenience, forcing the structure and layout of urban land use to be continuously adjusted and optimized to improve the ULUE. Thus, the following hypothesis is presented:

**H1.**  *The urbanization of the population can contribute to the increase in the ULUE*.

② Economic urbanization is the process of industrial agglomeration and benefit creation in cities. In economic terms alone, the continuous agglomeration of industries will generate economies of scale with strong positive externalities in most cases. During the development of economic urbanization, the industrial structure is continuously optimized and upgraded, resulting in improved resource allocation efficiency, lower pollutant emissions, and less undesired outputs. Different industries have different requirements for land, which will enable the optimization of urban land use layout and structure, thus improving the ULUE. Thus, the following hypothesis is presented:

**H2.**  *Economic urbanization can contribute to the increase in the ULUE*.

③ Spatial urbanization is the carrier of the N-TU, and the advancement of the urbanization process will definitely be reflected in space. Spatial urbanization includes the formation of urban carriers with the characteristics of modern civilization and the improvement of infrastructure such as the transportation conditions, reflecting the transformation of rural construction land into urban construction land and the improvement of spatial accessibility. On the one hand, the growth of the urban population and the expansion of the urban economy require urban land expansion, so the transformation of rural construction land into urban construction land is the inevitable result of urbanization development. Land is the carrier of urbanization development, and the integration and superposition of land as a production factor with other production factors helps to reduce the average production and management costs of products, thus increasing the economic benefits of urban land use. On the other hand, the urban transportation system is the carrier of the internal and external connectivity of cities, and the improvement in transportation and other infrastructure in the process of urbanization improves spatial accessibility. The improvement in spatial accessibility can shorten the distance between the extremities of satellite territories and major centers, alleviate the pressure of people living in the core areas of the city, encourage people to move to areas where they can afford the cost of living, and release land for more productive industrial sectors, thus optimizing the land use structure. In addition, improved spatial accessibility reduces the cost of land development and utilization in the suburbs, and some industries that are more sensitive to land rents will gradually move to suburban areas with lower rents due to improved accessibility, thus optimizing the land use layout. In general, spatial urbanization increases the economic benefits of urban land use, optimizes the structure and layout of urban land use, and, thus, improves the ULUE. Thus, the following hypothesis is presented:

**H3.**  *Spatial urbanization can contribute to the increase in the ULUE*.

④ Social urbanization reflects the process of the gradual equalizing of public services and infrastructure construction, which is led by the government and has strong positive externalities. Improving public services and infrastructure construction is beneficial to people’s psycho-physical wellbeing. It can also boost residents’ consumption, attract human capital, enhance labor productivity, and promote technological innovation, thus driving economic growth and increasing the economic value of land use. Meanwhile, the equalizing of public services and infrastructure construction can enable the rural migrant population to enjoy the same public resources as the urban population, create a good, harmonious, and stable social atmosphere, and enhance the social value of land use. Thus, the following hypothesis is presented:

**H4.**  Social urbanization can contribute to the increase in the ULUE.

### 3.3. Methodology

#### 3.3.1. Variables

##### Dependent Variable

The dependent variable in the study is the ULUE. Referring to the research results of Jiang et al. [49], Yu et al. [53], and Xue et al. [64], this study establishes a ULUE evaluation index system in terms of outputs (containing desired outputs and undesired outputs) and inputs. Specifically, the input indicators include land input, capital input, and labor input; output indicators include economic output, social output, desired environmental output, and undesired environmental output. Table 1 shows the detailed index system.

##### Independent Variable

The independent variable of this study is the N-TU(u). The N-TU is an integrated system involving the population, space, economy, and society in many aspects, emphasizing the coordinated development of all parts of urbanization. Thus, according to the principles of comprehensiveness, scientificity, systematization, and accessibility and with reference to the existing research results of Zhao et al. [38], Deng et al. [10] and Li et al. [40], the present study establishes an evaluation indicator system for the N-TU level, which contains four subsystems of spatial urbanization, urbanization of the population, economic urbanization, and social urbanization, with 11 specific indicators (Table 2).

##### Control Variables

Considering that many factors affect the ULUE, to prevent bias in the estimation results caused by the omission of relevant variables and to avoid the problem of multicollinearity with the indicators in the comprehensive index system of N-TU, four control variables were chosen.

① Degree of government intervention (gov). China’s economy is “government-led” in nature, and the government has a strong ability to intervene in social and economic development. Local governments can stimulate the economy through the introduction of relevant policies and large-scale investment activities, which can achieve the aggregation of production factors such as capital and labor in the short term and improve factor productivity, which in turn affects the ULUE [65].In addition, the government can also optimize the structure and layout of urban land use through land planning and other means to reduce the waste of idle urban land and, thus, improve the efficiency of urban land use. Referring to the practice of Zhao et al. [16], the degree of government intervention was measured using the proportion of local government fiscal expenditure in GDP.

② Environmental regulation (en). In the process of urban land use, people not only consume natural resources but also produce pollutants from human production, living, and other activities. Urban domestic waste in landfills and incineration produce toxic and harmful gases, with part of the gases released as well as other pollutants landing through rainfall onto the soil, causing harm to the soil environment, threatening urban land resources, and affecting the ULUE. Therefore, in the context of sustainable development, the degree of greening intensification will affect the level of urban land intensification. According to Porter’s hypothesis, reasonable and effective environmental regulation can stimulate enterprises to improve production technology and increase economic and technical efficiency, which in turn can improve urban land use efficiency. Referring to Hu et al. [66], the domestic waste disposal rate was selected as a substitute index for environmental regulation.

③ Degree of openness to the outside world (op). The knowledge and technical achievements brought by foreign investors, such as advanced technology and management experience, can prompt the government and urban land users to improve land use practices, which in turn can influence the ULUE. Referring to the study of Zhang et al. [67], we used the ratio of foreign direct investment to GDP to measure the degree of openness.

④ Science and technology level (tel). The improvement of the science and technology level can make the free flow of various factors of production subject to less resistance; so, the market regulation mechanism can be given greater play, and labor productivity can be increased. In addition, the improvement of science and technology level is conducive to the improvement of production technology and pollutant management level by enterprises, and the progress of production technology will promote the improvement of resource utilization efficiency, thus affecting the ULUE. Referring to the study of Wang et al. [68], the level of science and technology was measured by the total research and development expenditure.

#### 3.3.2. Models

Considering that the ULUE has a certain continuity in time, the magnitude of the ULUE in the current period may be influenced by the previous period. Thus, a dynamic model was set by adding the first-order lag term of the ULUE to the econometric model. The benchmark model of the impact of the N-TU on ULUE is shown as follows:

(1) $$ulue_{i,t}=\beta_{0}+\beta_{1}ulue_{i,t-1}+\beta_{2}u_{i,t}+\sum_{v=1}^{n}\rho_{v}X_{v,i,t}+\mu_{i}+\varepsilon_{it},$$

where *i* and *t* represent the region and year, respectively; *v* represents the number of control variables; $\beta_{0}$ is a constant term; $\beta_{1}$, $\beta_{2}$, and $\rho_{v}$ are coefficients; $ulue_{i,t}$ denotes the ULUE of region *i* in year *t*, $ulue_{i,t-1}$ indicates the ULUE in year *t* − 1; $u_{i,t}$ represents the N-TU level; *X* means the control variable; $\varepsilon_{it}$ is the random disturbance term; and $\mu_{i}$ denotes the region fixed effect.

To explore deeply the ways in which new urbanization affects the ULUE, we further discuss how new urbanization affects the ULUE through various subsystems. The following four models were established:

(2) $$ulue_{i,t}=\beta_{0}+\beta_{1}ulue_{i,t-1}+\beta_{2}pu_{i,t}+\overset{n}{\underset{v=1}{\sum }}\rho_{v}X_{v,i,t}+\mu_{i}+\varepsilon_{it}$$

(3) $$ulue_{i,t}=\beta_{0}+\beta_{1}ulue_{i,t-1}+\beta_{2}eu_{i,t}+\overset{n}{\underset{v=1}{\sum }}\rho_{v}X_{v,i,t}+\mu_{i}+\varepsilon_{it}$$

(4) $$ulue_{i,t}=\beta_{0}+\beta_{1}ulue_{i,t-1}+\beta_{2}lu_{i,t}+\overset{n}{\underset{v=1}{\sum }}\rho_{v}X_{v,i,t}+\mu_{i}+\varepsilon_{it}$$

(5) $$ulue_{i,t}=\beta_{0}+\beta_{1}ulue_{i,t-1}+\beta_{2}su_{i,t}+\overset{n}{\underset{v=1}{\sum }}\rho_{v}X_{v,i,t}+\mu_{i}+\varepsilon_{it}$$

where $su_{i,t},$ $pu_{i,t},lu_{i,t}$ and $eu_{i,t}$ indicate the level of social urbanization, economic urbanization, urbanization of the population, and spatial urbanization, respectively. The other variables have the same meanings as Model 1. Table 3 demonstrates the descriptive statistics for each variable.

#### 3.3.3. Methods

##### Entropy Weight Method

In this study, in order to avoid the effect of the subjective weighting method on the precision of evaluation results and the problem that the comprehensive index measurement is not comprehensive because of the elimination of indicators with a low contribution rate by objective weighting methods, such as factor analysis and principal component analysis, therefore, the study used the entropy weight method [69] to calculate the N-TU level based on the comprehensive evaluation indicator system of the N-TU. The entropy weight method is an objective weighting method, which uses the magnitude of information provided by the entropy value of each indicator to determine the weight of the indicator. By normalizing the original indicators and defining the weights [70], the objectivity and comprehensiveness of the calculated results are ensured. Similarly, the levels of social urbanization, economic urbanization, the urbanization of the population, and spatial urbanization were calculated using the entropy weight method. The calculation process of the method is as follows.

① Standardization of the original indicators:

Positive indicators:

(6) $$X_{ij}=\frac{x_{ij}-x_{jmin}}{x_{imax}-x_{jmin}}$$

Negative indicators:

(7) $$X_{ij}=\frac{x_{jmax}-x_{ij}}{x_{jmax}-x_{jmin}}$$

where $x_{ij}$ is the original data, and $X_{ij}$ denotes the standardized value $\left(i=1,2\ldots ,m;j=1,2\ldots ,n\right)$.

② Calculate the proportion of the *j*th indicator:

(8) $$P_{ij}=\frac{X_{ij}}{\sum_{i=1}^{m}X_{ij}}$$

③ Calculate the entropy value of the *j*th indicator:

(9) $$E_{j}=-\frac{1}{\ln\left(m\right)}\overset{m}{\underset{i=1}{\sum }}(P_{ij}\times lnP_{ij})$$

④ Calculate the weight of the *j*th indicators:

(10) $$W_{j}=\frac{1-E_{j}}{\sum_{j=1}^{n}\left(1-E_{j}\right)}$$

⑤ Calculate the comprehensive evaluation indexes for N-TU and its subsystems:

(11) $${\mathrm{Index}}_{i}=\overset{n}{\underset{j=1}{\sum }}W_{j}X_{ij}$$

##### The SE-SBM Model Considering Undesirable Outputs

The city is a complex system. This study attempts to avoid the issue of incorrectly setting the form of the production function because of poor consideration. Thus, this study referred to the existing research results [71] and chose the DEA to measure the ULUE, a representative method of nonparametric methods that do not require a specific production function to be set in advance.

The DEA is a nonparametric approach put forward by Charnes and Cooper [72] in 1978 to assess the effectiveness of DMUs (Decision Making Units) using mathematical planning models. The traditional DEA method uses radial adjustment when adjusting the data, and the measured results are somewhat different from the actual ones. Furthermore, the existence of the input redundancy and output insufficiency is ignored, impacting the accuracy of the measured results. Tone [73] presented an SBM model containing slack variables. Assuming that each DMU has *m* input vectors, *s* output vectors, and *n* DMUs, $x\in R_{m}$ and $y\in R_{s}$ define the matrices *X* and *Y* as $X=\left(x_{ij}\right)\epsilon R_{m\times n}$ and $Y=\left(y_{ij}\right)\epsilon R_{s\times n}$, respectively. Assuming that X>0 and Y>0, the production possibilities set is $P=\left\{\left(x,y\right)\left|x\geq X\lambda ,y\leq Y\lambda ,\lambda \geq 0\right.\right\}$. The SBM model can be specifically expressed as follows:

(12) ρ∗=min1−1m∑i=1mSi−xi01+1s∑r=1sSr+yr0, s.t.X0=Xλ+S−;y0=Yλ−S+S−≥0,S+≥0,λ≥0,

where $\rho^{*}$ denotes the efficiency value of the DMU, which takes values in the range of 0–1. λ indicates the nonnegative intensity vector, $S^{-}$ is the slack variable for inputs, and $S^{+}$ is the slack variable for outputs.

Given that the traditional DEA can only distinguish whether the DMUs are efficient or not, it cannot be compared and ranked. Thus, the SE-SBM model was proposed, solving this problem. The principle of the model is to exclude a DMU when evaluating its efficiency and replace it with a linear combination of inputs and outputs from other DMUs. When an efficient DMU increases its inputs proportionally, its efficiency value can be kept constant, and its super-efficient evaluation value is the proportional increase in its inputs. Specifically, the SE-SBM model can be expressed as follows:

(13) ρ∗=min1+1m∑i=1mSi−xi01−1s∑r=1sSr+yr0,s.t.X0≥Xλ−S−Y0≤Yλ+S+S−≥0,S+≥0,λ≥0

The SE-SBM model can well overcome the limitations of the traditional SBM and DEA. Considering that negative externalities occur in the land use process, this article adopted the SE-SBM model with undesirable outputs [53,74] to estimate the ULUE of the YREB from 2011 to 2020. Assuming that each DMU has *m* input vectors, *n* DMUs, $s_{1}$ desired output vectors, and $s_{2}$ undesired output vectors, $x\in R_{m}$, $y^{g}\in R_{s_{1}}$, and $y^{b}\in R_{s_{2}}$ define the matrices *X*, $Y^{g}$, and $Y^{b}$ as $X=\left(x_{ij}\right)\epsilon R_{m\times n}$, $Y^{g}=\left(y_{ij}^{g}\right)\epsilon R_{s_{1}\times n}$, and $Y^{b}=\left(y_{ij}^{b}\right)\epsilon R_{s_{2}\times n}$, respectively. Assuming that X>0, $Y^{g}>0$, and $Y^{b}>0,$ the model can be formulated as follows:

(14) ρ∗=min1m∑i=1mx¯xi01s1+s2(∑r=1s1yg¯yr0g+∑u=1s2yb¯yr0b),              s.t.x¯≥Xλyg¯≤Ygλyb¯≥Ybλλj≥0,j=1,2,…,nx¯≥x0,i=1,2,…,myg¯≤y0g,r=1,2,…,s1yb¯≥y0b,u=1,2,…,s2,

where $\rho^{*}$ indicates the ULUE of the YREB, $\bar{x}$ is the slack variable for the inputs, $\bar{y^{g}}$ is the slack variable for the desired outputs, and $\bar{y^{b}}$ is the slack variable for the undesired outputs.

##### Sys-GMM

Considering that the ULUE of the previous period may have an impact on the ULUE of the current period, the first-order lag term of the ULUE ($ulue_{i,t}$) was included in the model. Therefore, the parameter estimation methods for static panel models, such as mixed OLS estimation, fixed effects, or random effects estimation are not applicable to this model. Furthermore, endogeneity problems may be encountered in the parameter estimation for the above five models. Two approaches to parameter estimation for dynamic panel models, differential GMM (Dif-GMM) [75] and Sys-GMM [76], can overcome the endogeneity problem. Sys-GMM is more applicable to this study than Dif-GMM because of the following two reasons. Firstly, the sample of this study provides short panel data, and Sys-GMM is suitable for parameter estimation of short panel data. Secondly, Sys-GMM connects the difference equation and the original horizontal equation and uses the lagged term of the dependent variable and the first-order difference as instrumental variables, which improves the weak instrumental variable problem that may result from Dif-GMM and enhances estimation efficiency. Thus, Sys-GMM was selected to estimate the model parameters in this study.

## 4. Results

Based on the materials and methodology in Section 3, Section 4 investigates the evolutionary trends of the N-TU level and the ULUE in the YREB. In addition, the impact of the N-TU on ULUE is further investigated using the Sys-GMM method. Its purpose is to understand the current situation and evolutionary trends of the N-TU and ULUE and clarify the relationship between them, thus helping decision makers in the assessment and design of policy recommendations.

### 4.1. Evolution Trend of the N-TU Level

**Figure 3:**
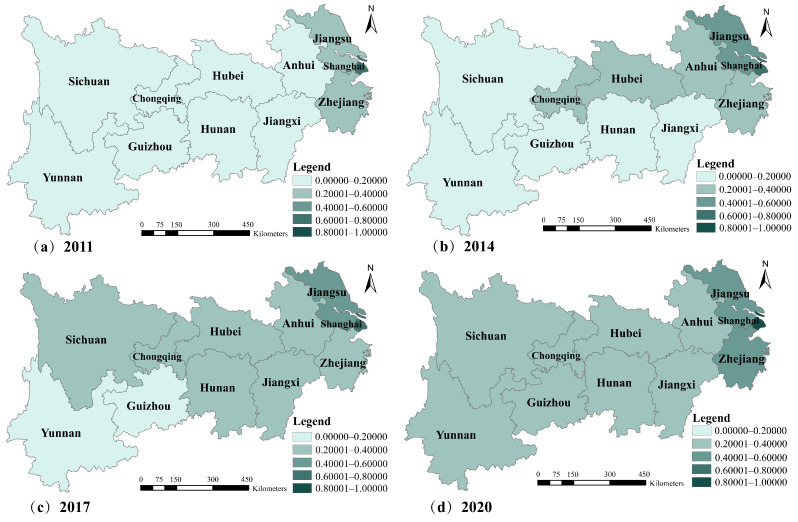
The spatial evolution pattern of the N-TU in the YREB, 2011–2010. **Source:** Authors’ drawing using ArcGIS10.7 software.

Based on the N-TU level evaluation indicator system and the calculation method mentioned above, the N-TU level of the YREB from 2010 to 2020 was obtained. This study used ArcGIS10.7 software to visualize the spatial distribution pattern of the N-TU level in various provinces and cities more intuitively. Given space limitations, four periods of 2011, 2014, 2017 and 2020 were selected for presentation (Figure 3).

**Figure 4:**
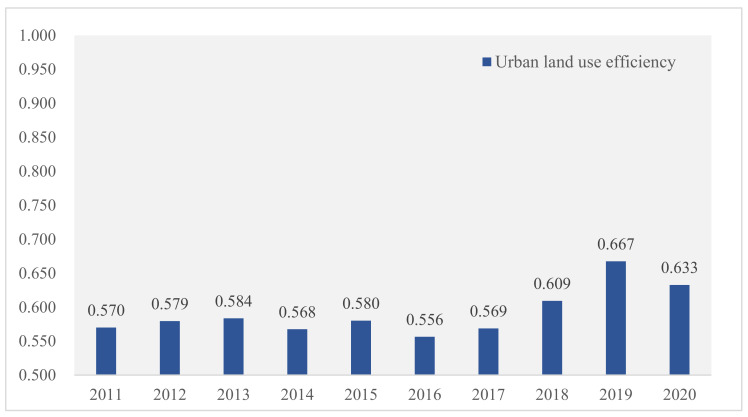
Evolution trend of the ULUE from 2011 to 2020. **Source:** Authors’ drawing.

The N-TU level of the YREB as a whole and each province (city) has exhibited a gradual upward trend from 2011 to 2020. Figure 4 shows that the N-TU level of the YREB had the characteristics of uneven spatial distribution across provinces, showing a decreasing pattern of downstream–midstream–upstream hierarchy, indicating that the development of the N-TU was unbalanced and insufficient in the YREB. The strategic goal of regional coordinated development has not yet been achieved. The main reason is the large differences in geographical location, economic conditions, human resources, and technological level among the upstream, midstream, and downstream regions of the YREB. Specifically, Shanghai’s N-TU level has been far ahead of other regions for a decade, reaching 0.8728 in 2020, with Jiangsu and Zhejiang following closely behind. As the national economic, trade, and financial center, Shanghai has been attracting talent and industry. The resulting agglomeration effect accelerated N-TU development in all aspects and drove the growth of surrounding areas. Furthermore, with the national policy tilting toward the upstream and midstream regions of the YREB, the gap between the N-TU levels of each region is gradually decreasing.

### 4.2. Evolution Trend of the ULUE

Based on the ULUE evaluation index system and using the SE-SBM model with undesirable outputs and Matlab R2016a software, the ULUE of the YREB from 2011 to 2020 was calculated. The results are shown in Figure 4.

From 2011 to 2020, the overall ULUE of the YREB exhibited a fluctuating upward trend, with prominent stage characteristics. The whole period from 2011 to 2016 was dominated by volatility. The ULUE increased steadily from 2011 to 2013 and fluctuated from 2014 to 2016. The fluctuation was caused by the belt and Road Strategy put forward in 2013, which increased the input into the Shanghai, Zhejiang, Chongqing, and Yunnan provinces (cities). However, the output did not appear in time, resulting in the decline of the ULUE in 2014, and the fluctuation in 2016 was affected by the global economic downturn. From 2016 to 2019, the ULUE showed a significant increasing trend. The reason for this trend is that, since the adoption of the Outline of the YREB Development Plan in 2016, provinces (cities) have started to focus on protecting the ecological environment, and urban pollutant emissions have been significantly reduced. Furthermore, government departments again emphasized the importance of ecological construction in 2018. They proposed that the YREB should be led by ‘ecological priority and green development’ for high-quality development. Governments paid greater attention to environmental protection. Under certain input factors, the high-quality development model of minimizing ecological and environmental losses in exchange for maximizing positive integrated land output has been continuously explored, thus increasing the ULUE year by year during this period. The decline in 2020, compared with that in 2019, was mainly because of the tertiary industries such as catering and tourism that were hit hard by the COVID-19 pandemic and secondary industries such as manufacturing and construction that were also affected to some extent because of the short-term shutdown, resulting in a significant decline in urban economic output and a decline in the ULUE.

### 4.3. Overall Impact Results Analysis

**Figure 5:**
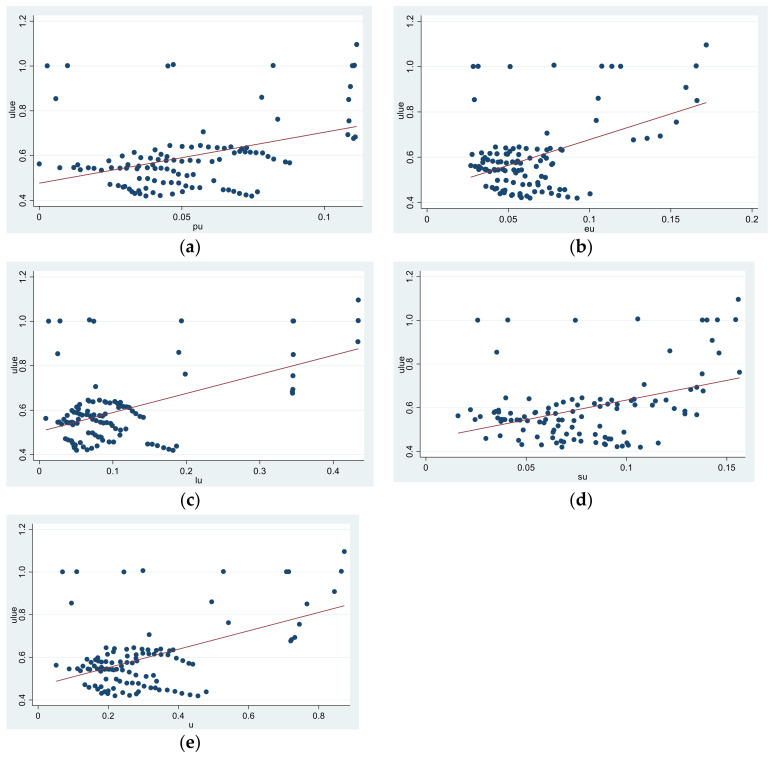
The linear fitting results of the relationship between the N-TU, its subsystems, and ULUE. (**a**) The urbanization of the population and ULUE. (**b**) Economic urbanization and ULUE. (**c**) Spatial urbanization and ULUE. (**d**) Social urbanization and ULUE. (**e**) N-TU and ULUE. **Source:** Authors’ drawing using the StataMP 16 software.

According to the internal mechanism analysis of the impact of the N-TU on the ULUE in Section 3.2, this study used StataMP 16 software to draw a linear scatter fitting graph to preliminarily judge the relationship between the N-TU and ULUE. Figure 5 shows that the N-TU and its subsystems positively affected the ULUE, which preliminarily confirms Hypotheses 1–4.

We used the dynamic panel regression model to further study the relationship between N-TU and ULUE in detail. A unit root test was conducted for the stationarity of the panel data before the regression of the dynamic panel regression model to avoid the problem of ‘pseudo-regression’ from affecting the reliability of the research findings. We adopted the most commonly used Levin-Lin-Chu method to examine the panel data. The test results presented in Table 4 indicate that all variables in the five models passed the unit root test. Therefore, dynamic panel regressions were performed on the panel data.

When using the Sys-GMM for dynamic panel regression, it is necessary to test the autocorrelation of the random disturbance term of the model and the validity of the instrumental variables. The AR (1) test and the AR (2) test were used to test the autocorrelation of the random disturbance term after difference. The original hypothesis was that there was no first-order autocorrelation and second-order autocorrelation in the random perturbation term. The Sargan test was used to determine the effectiveness of the instrumental variables, and the original hypothesis was that the selected instrumental variables were valid. As can be seen from Table 5, the *p*-value of the AR (1) test is 0.083, and the original hypothesis was rejected at the 10% significance level, indicating that the random disturbance term had first-order autocorrelation. The *p*-value of AR (2) test was 0.619, which accepts the original hypothesis, indicating that there was no second-order autocorrelation in the random disturbance term. The *p*-value of the Sargan test was 0.154, which was greater than 0.1, and this test accepted the original hypothesis, indicating that the instrumental variables were valid. In summary, the model (1) passed the Sargan test, AR (1) test, and AR (2) test. Therefore, the estimation of the parameters of Model (1) using the Sys-GMM was appropriate, and the parameter evaluation results are reliable.

The model (1) was evaluated using the Sys-GMM estimation and Stata 16.0 software to investigate the impact of the N-TU on the ULUE in the YREB. The results were obtained, as shown in Table 5. The findings showed that the first order lagged term of the ULUE had a coefficient of 0.5495 and passed the significance test at the 1% level, indicating that the ULUE of the YREB had large inertia during the study period. The ULUE in the former stage had a significant positive influence on the ULUE in the present stage, and the higher the ULUE in the previous period, the higher the ULUE in the current period, indicating that the enhancement of the ULUE has a cumulative effect. The coefficient of the N-TU was 0.3816 and passed the test, showing that N-TU had a positive influence on the ULUE, and the improvement in the N-TU level promoted the improvement of the ULUE.

For the control variables, the coefficient of government intervention degree (gov) was 0.2805, indicating that government intervention positively affected the ULUE. Government measures, such as guiding the gathering of labor, capital, and other production factors and optimizing the land use structure, can promote the increase in the ULUE. The environmental regulation (en) was significantly negative at the 1% level, showing that the level of environmental regulation in the YREB has not yet led to an increase in the ULUE. The current level of environmental regulation has not achieved the expected positive impact, and the effectiveness of environmental regulation is very limited [77]. Similarly, Xue et al. [64] found that environmental regulation also had a negative impact on urban land use efficiency of cities in the Yellow River Basin. The estimated coefficient of the technology level (tel) was −0.0240 and passed the test, signifying that it was negatively correlated with ULUE. This result may be because enterprises invest a lot of money in science and technology research and development (R&D), which squeezes production costs and reduces operating profits. Furthermore, scientific and technological R&D has a large investment and long return cycle. Thus, the marginal effects of scientific and technological R&D do not appear in the short term. The coefficient of the extent of external openness (op) was −1.3121, indicating that the degree of openness to the outside world has not yet contributed to the improvement of the ULUE.

### 4.4. Subsystem Impact Results Analysis

This study conducted a Sys-GMM estimation of Models 10–13 using Stata 16.0 software to explore the impact of four subsystems of the N-TU on the ULUE and discuss further the specific impact path of the N-TU on the ULUE in the YREB. Table 6 displays the estimation results.

After the Sargan test and the AR test, the regression models did not have the problem of second-order sequence autocorrelation and overidentification, indicating that the results of each model were reliable. The empirical results in Table 6 show that the first-order lagged term coefficients of the ULUE in regression models (2)–(5) were 0.5872, 0.5497, 0.5571, and 0.5718, respectively. They all passed the significance test at the 1% level, indicating that the ULUE in the former stage had a significant positive influence on the ULUE in the present stage, which again proves that ULUE has a significant cumulative effect. The urbanization of the population coefficient was 1.8720, indicating that the urbanization of the population had a significant positive influence on the ULUE during the study period. The improvement of the level of the urbanization of the population will facilitate the improvement of the ULUE. This conclusion confirmed Hypothesis 1 of this paper. The coefficient of economic urbanization had a positive significance at the 1% level. Every 1% growth in economic urbanization caused a 2.0415% increase in the ULUE, indicating that economic urbanization positively impacted ULUE. The increase in the economic urbanization level will promote the increase in the ULUE. This conclusion confirmed Hypothesis 2 of this paper. The spatial urbanization coefficient was 0.6260, indicating that spatial urbanization had a significant positive effect on the ULUE. The increase in the spatial urbanization level will promote the improvement of the ULUE. This conclusion confirmed Hypothesis 3 of this paper. The social urbanization coefficient was 1.6600. Therefore, the increase in the social urbanization level will significantly improve the ULUE. This conclusion confirmed Hypothesis 4 of this paper. Furthermore, from the estimated coefficient magnitude, economic urbanization had the greatest contribution to the ULUE.

### 4.5. Robustness Examination

Two approaches were applied in this study to verify the robustness of the regression results to ensure the accuracy and stability of the empirical test results.

1. We removed the control variables. The model was re-estimated by removing the data from the control variables, and the outcomes are presented in Table 7. Compared with the previous empirical results, the model passed the Sargan and AR test, and the direction of the main variables remained unchanged.
2. We replace a control variable. The domestic waste treatment rate variable, which indicated the environmental factor, was replaced with the amount of industrial pollution control investment, and its logarithm was taken as the variable. Table 8 presents the test results. Compared with the results of the previous empirical tests, the models passed the Sargan and AR test, the direction of the main variables did not change, and the significance passed the test. In conclusion, the above two methods indicate that the empirical findings of the article are robust.

## 5. Discussion and Policy Implications

### 5.1. Discussion

This study systematically examined the evolutionary trends of the N-TU and the ULUE, and the relationship between them for the YREB from 2011 to 2020. In view of the four questions raised in the Section 1, we provided the answers through empirical research. ① The N-TU level for 2011–2020 was obtained by constructing a comprehensive N-TU evaluation index system and applying the entropy weight method. The study found that the overall N-TU level showed a gradual increase, which was mainly due to the national and local governments investing resources to vigorously promote the construction of the N-TU. In addition, the N-TU development in the YREB was characterized by uneven spatial distribution across provinces, mainly due to the large differences in human, material, and financial resources among the provinces in the YREB. In recent years, the gap in the N-TU level among regions has gradually narrowed. ② By constructing a comprehensive evaluation index system of the ULUE, the ULUE was calculated from 2011 to 2020 using the SE-SBM model. The results showed that the overall ULUE of the YREB showed a fluctuating upward trend with obvious phase characteristics. ③ The effect of the N-TU on the ULUE was investigated by using the Sys-GMM estimation method. It was shown that the N-TU had a positive effect on the ULUE. In the process of N-TU, the economic and social benefits of urban land use were increased by revitalizing idle residential bases in rural areas and optimizing the land use structure, thus improving the ULUE. ④ The effect of the four subsystems of the N-TU on the ULUE was investigated using the Sys-GMM estimation method. The empirical results showed that the four subsystems of the N-TU had positive effects on the ULUE. The ULUE can be promoted by enhancing the level of spatial urbanization, the urbanization of the population, economic urbanization, and social urbanization.

This paper did have some limitations. The basic national conditions of China and developed countries are quite different, the urbanization level of developed countries is much higher than that of China, and some developed countries are vast and sparsely populated; so, the research results may not be applicable to those developed countries at present. However, there are still many developing countries around the world, such as countries along the “Belt and Road” and other low-income countries, which are still trying to promote urbanization, and the contradiction between land supply and demand still exists. Therefore, in the process of urbanization, it is necessary to actively explore ways to improve the ULUE. In this paper, a set of systematic research methods was formed by studying the N-TU and ULUE in the YREB, which can provide references for other countries or economies to study similar issues.

### 5.2. Policy Implications

On the basis of the empirical results, the relevant departments should actively promote the construction of the N-TU and actively promote social urbanization, economic urbanization, spatial urbanization and urbanization of the population to increase the ULUE.

① They should actively promote the urbanization of the population and focus on its role in improving the ULUE. The government should continue to deepen the household registration reform system, as this is the root system that hinders the citizenship of the rural transfer population. In addition, it should improve the housing security system and employment system for the rural transfer population, as this is the basis for the rural transfer population to be able to live in the city.

② They should actively promote economic urbanization and focus on its role in improving the ULUE. Local enterprises should strengthen exchanges and cooperation to boost the transformation and upgrading of industrial structure, which will promote the coordinated development of the regional economy and reduce the undesired outputs.

③ They should actively promote spatial urbanization and give focus on its role in improving the ULUE. The government should promote the implementation of the policy of linking urban and rural construction land increases and decreases to optimize the allocation of regional land resources. At the same time, it should strengthen intra-city as well as inter-city transportation to improve spatial urbanization, thus enhancing land use efficiency.

④ They should actively promote social urbanization and focus on its role in improving the ULUE. The government should improve the population residence permit system, and strengthen the education and medical security of the rural migrant population to facilitate the improvement of the ULUE.

## 6. Conclusions

This study used panel data from 2011 to 2020 for 11 provinces (cities) in the YREB. It constructed a composite evaluation indicator system of the N-TU and ULUE and adopted the SE-SBM model with undesirable outputs and the entropy weight method to evaluate the ULUE and N-TU, respectively. On the basis of analyzing the impact mechanism, a dynamic panel regression model was constructed, and the Sys-GMM method was used to study the impact of the N-TU on the ULUE. The core findings of the study were as follows. (1) During the study period, the level of the N-TU in the YREB showed an increasing trend on the whole and had the characteristic of uneven inter-provincial spatial distribution. The N-TU level in the downstream regions was higher than in the upstream and midstream regions. (2) The ULUE of the YREB grew from 0.5700 in 2011 to 0.6327 in 2020, showing a fluctuating upward trend as a whole during the studied period. (3) The N-TU had a significant positive influence on the ULUE. Every 1% increase in the N-TU level increased the ULUE by 0.3816%. The estimated coefficients of social urbanization, economic urbanization, spatial urbanization, and the urbanization of the population were 1.6600, 2.0415, 0.6260, and 1.8720, respectively, all of which passed the significance test, showing that each subsystem of the N-TU had a positive influence on the ULUE.

This study considered that the ULUE in the previous period may have an impact on the ULUE in the current period; we investigated the impact of the N-TU on the ULUE using a dynamic panel regression model, which will help policy makers to understand the current status of the development of the N-TU and ULUE comprehensively and objectively, as well as to grasp the paths that promote the improvement of the ULUE. Importantly, the methodology and structure used in this paper can provide a reference for related studies in this field. However, there are gaps in this study that should be filled through further research. First, the data can be further refined, and exploring the impact of the N-TU on the ULUE based on city-level panel data can provide a more detailed understanding of the relationship between the two, thus providing reasonable policy recommendations for policymakers based on local conditions. In addition, this study cannot verify the existence of spatial spillover effects, which can be further investigated by using spatial lag models in future studies.

## Figures and Tables

**Table 1:** Evaluation index system of the ULUE.

Target Layer	Subsystem Layer		Indicator Layer
**Urban land use efficiency**	Input indicators	Land input	Urban construction land area (km^2^)
		Capital input	Fixed assets investment amount (CNY billion)
		Labor input	Number of employees in secondary and tertiary industries (10,000 people)
	Desired output indicators	Economic output	Secondary and tertiary industries added value (CNY billion)
		Social Output	Average wage of urban residents (CNY)
		Environmental Output	Green space per capita (m^2^/people)
	Undesirable output indicators	Undesired environmental outputs	Industrial wastewater discharges (10,000 tons)
			Industrial SO_2_ dioxide emissions (ton)
			Industrial smoke (dust) emissions (ton)

**Table 2:** Evaluation index system of the N-TU.

Target Layer	Subsystem Layer	Indicator Layer	Indicator Attributes	Unit	Weights
New-type urbanization	Urbanization of the population (0.112)	The proportion of employed persons in secondary and tertiary industries to all employed persons	+	%	0.394
		Urban population as a proportion of total resident population	+	%	0.606
	Economic urbanization (0.182)	Non-agricultural GDP per unit of built-up area land	+	billion/km^2^	0.400
		Value added of tertiary industry as a proportion of GDP	+	%	0.110
		Per capita social consumption	+	CNY 10,000/person	0.490
	Spatial urbanization (0.497)	Urban road area per capita	+	m^2^/person	0.116
		The proportion of the built-up area to the total area of the jurisdiction	+	%	0.771
		Built-up area per capita	+	km^2^/10,000 people	0.113
	Social urbanization (0.210)	Number of public transportation vehicles per 10,000 people	+	Vehicles/10,000 people	0.574
		The proportion of educational expenditure in fiscal expenditure	+	%	0.184
		Number of beds in medical institutions per 10,000 people	+	Beds/person	0.242

Note: “+” indicates a positive indicator.

**Table 3:** Descriptive statistics of variables.

Variable Abbreviation	Variable Name	Mean	Std. Dev	Min	Max	Sample Size
ulue	Urban land use efficiency	0.5966	0.1583	0.4192	1.0961	110
u	New-type urbanization	0.3039	0.1783	0.0515	0.8728	110
pu	Urbanization of the population	0.0527	0.0266	0.0001	0.1113	110
eu	Economic urbanization	0.0643	0.0319	0.0269	0.1718	110
lu	Spatial urbanization	0.1083	0.0939	0.0087	0.4340	110
su	Social urbanization	0.0787	0.0350	0.0160	0.1563	110
gov	Degree of government intervention	0.2236	0.0579	0.1206	0.4022	110
en	Environmental factor	0.9822	0.0307	0.8272	1.0000	110
lntel	Science and technology level	5.8705	1.0596	3.5921	7.8038	110
op	Extent of openness to the external world	0.0230	0.1182	0.0017	0.0455	110

**Table 4:** Unit root test result.

Variable	Statistics	*p*-Value	Result
ulue	−11.1388	0.0000	stable
u	−1.7502	0.0400	stable
pu	−2.0725	0.0191	stable
eu	−2.0117	0.0221	stable
lu	−2.2544	0.0121	stable
su	−6.5455	0.0000	stable
gov	−1.8990	0.0288	stable
en	−4.9358	0.0000	stable
lntel	−4.5870	0.0000	stable
op	−1.9510	0.0255	stable

Note: This unit root test contains individual fixed effect terms and trend terms.

**Table 5:** Regression results of model (1).

Variable	Coefficient	*t*-Value	*p*-Value
uluei-1	0.5495	4.30	0.002 ***
u	0.3816	2.12	0.000 ***
gov	0.2805	1.02	0.033 **
en	−0.0189	−4.06	0.007 ***
lntel	−0.0240	−2.33	0.093 *
op	−1.3121	−3.42	0.006 ***
Constant term	0.2830	5.71	0.005 ***
AR (1) test	*p* = 0.083		
AR (2) test	*p* = 0.619		
Sargan test	*p* = 0.154		

Note: ***, ** and * indicate significant at the 1%, 5% and 10% levels, respectively.

**Table 6:** Results of the impact of the N-TU subsystems on the ULUE.

Variable	Model (2)	Model (3)	Model (4)	Model (5)
uluei-1	0.5872 ***	0.5497 ***	0.5571 ***	0.5718 ***
	(4.16)	(4.38)	(4.16)	(4.03)
pu	1.8720 ***			
	(3.31)			
eu		2.0415 ***		
		(4.25)		
lu			0.6260 ***	
			(3.63)	
su				1.6600 *
				(1.90)
Constant term	0.1249 **	0.4963 *	0.1336 ***	0.3283 *
	(2.27)	(1.93)	(3.36)	(1.63)
Control variables	YES	YES	YES	YES
AR (1) test	*p* = 0.080	*p* = 0.084	*p* = 0.081	*p* = 0.081
AR (2) test	*p* = 0.605	*p* = 0.633	*p* = 0.612	*p* = 0.611
Sargan test	*p* = 0.152	*p* = 0.110	*p* = 0.169	*p* = 0.100

Note: ***, ** and * indicate significance at the 1%, 5%, and 10% levels, respectively; *T*-values in parentheses.

**Table 7:** Robustness Test—Removal of the Control Variables.

Variable	Model (1)	Model (2)	Model (3)	Model (4)	Model (5)
uluei-1	0.5759 ***	0.5909 ***	0.5916	0.5762 ***	0.5769 ***
	(4.43)	(4.18)	(4.51)	(4.31)	(4.39)
u	0.1864 *				
	(2.10)				
pu		0.8124			
		(1.20)			
eu			1.1355 **		
			(2.53)		
lu				0.3599 **	
				(2.76)	
su					0.7394
					(1.15)
Control variables	/	/	/	/	/
Constant term	0.2009 **	0.2066 *	0.1746 **	0.2193 **	0.1979 **
	(2.56)	(2.14)	(2.27)	(2.70)	(2.52)
AR (1) test	*p* = 0.083	*p* = 0.079	*p* = 0.085	*p* = 0.082	*p* = 0.082
AR (2) test	*p* = 0.619	*p* = 0.611	*p* = 0.620	*p* = 0.614	*p* = 0.617
Sargan test	*p* = 0.226	*p* = 0.221	*p* = 0.190	*p* = 0.217	*p* = 0.224

Note: ***, ** and * indicate significance at the 1%, 5%, and 10% levels, respectively; *T*-values in parentheses.

**Table 8:** Robustness test—A Control Variable Replaced.

Variable	Model (1)	Model (2)	Model (3)	Model (4)	Model (5)
ulue_i-1_	0.5679 ***	0.6128 ***	0.5903 ***	0.5653 ***	0.5920 ***
	(4.80)	(5.03)	(5.51)	(4.33)	(5.00)
u	0.3699 ***				
	(3.95)				
pu		1.8774 **			
		(2.43)			
eu			2.0229 ***		
			(4.94)		
lu				0.5923 **	
				(2.90)	
su					1.8556 **
					(2.34)
Control variables	YES	YES	YES	YES	YES
Constant term	0.2042 **	−0.0789 **	0.2270 *	0.1764 *	0.1093 *
	(2.51)	(−1.61)	(1.97)	(1.97)	(1.76)
AR (1) test	*p* = 0.089	*p* = 0.088	*p* = 0.091	*p* = 0.085	*p* = 0.088
AR (2) test	*p* = 0.615	*p* = 0.600	*p* = 0.621	*p* = 0.609	*p* = 0.613
Sargan test	*p* = 0.163	*p* = 0.159	*p* = 0.111	*p* = 0.164	*p* = 0.120

Note: ***, ** and * indicate significance at the 1%, 5%, and 10% levels, respectively; T-values in parentheses.

## Data Availability

The data are contained within the article.
